# 329. Severe Obesity: A Critical Risk Factor for In-Hospital Complications and Mortality in the Hispanic Population

**DOI:** 10.1093/ofid/ofab466.531

**Published:** 2021-12-04

**Authors:** Moulika Baireddy, Sivaram Neppala, Dinesh Kumar Sundarakumar, Hector Santos

**Affiliations:** TIGMER/Laredo Medical Center, Laredo, Texas

## Abstract

**Background:**

Obesity, Diabetes mellitus type 2, race and other characteristics has been associated with an increased risk of adverse outcomes in patients with COVID-19 disease. The prevalence of obesity in the United States in 2017-2018 was 42.4%. Webb County, Texas with a 95.6% Hispanic population shows an obesity prevalence of 35.8% in 2014. It is unclear whether obesity increases the risks of complications and mortality in Hispanic population from COVID-19 disease.

**Methods:**

This is a retrospective cohort study of patients admitted to the hospital with the diagnosis of COVID-19 between March 2020 and August 2020. 950 patients were tested and admitted to the hospital with the diagnosis of COVID-19 pneumonia. Patients were categorized into classes of body habitus by BMI: underweight (< 18.5), normal (18.5-24.9), overweight (25.0-29.9), obesity class 1 (30.0-34.9), obesity class 2 (35.0-39.9), and obesity class 3 ( >40.0).

**Results:**

950 Hispanic patients were included (Male-52.8%, Female- 47.2%) in the study. In total, 19.05% of our patients died during the hospitalization with an increased risk of mortality in patients having obesity class 2 (RR 4.14, 95% CI = 2.2–7.7 p=< 0.0001), and obesity class 3 (RR 6.0, 95% CI = 1.3–4.6 p=< 0.0001) compared with those with normal BMI. Mortality was higher in obese patients who required invasive mechanical ventilation at 93.75% compared to obese patients who were non-ventilated at 4.29%. Patients with obesity class 2 and 3 had higher risks of in-hospital complications including AKI requiring renal replacement therapy, ARDS, and arrythmias most commonly atrial fibrillation/flutter at 26.7%, 18.42% and 13.5%.

Characteristics of In-hospital complication complications due to COVID-19 disease

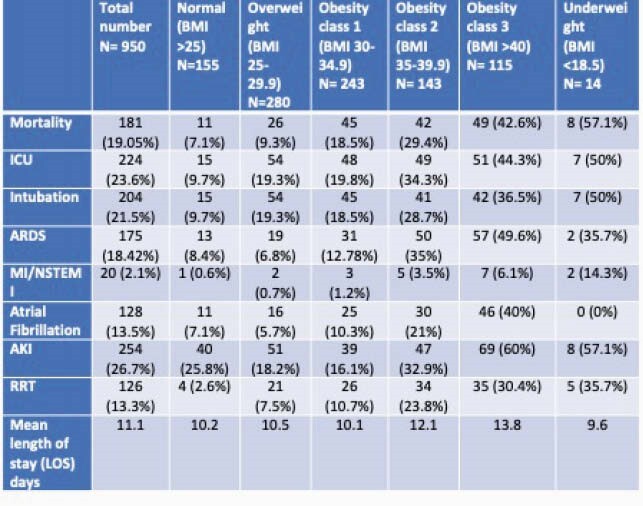

**Conclusion:**

Patients admitted to the hospital with the diagnosis of COVID-19 disease with obesity classes 2 and 3 have a significantly increased risk of mortality as compared to normal and overweight patients. Severe obesity was also associated with increased hospital complications of AKI, ARDS, and Atrial Fibrillation. This further affirms that obesity is a pertinent risk factor to be considered in COVID-19 patients.

**Disclosures:**

**All Authors**: No reported disclosures

